# GABA receptor associated protein changes the electrostatic environment around the GABA type A receptor

**DOI:** 10.1002/prot.26241

**Published:** 2021-10-03

**Authors:** Benedict W. J. Irwin, Clara C. Wanjura, Daniel Molnar, Michael J. Rutter, Michael C. Payne, P.‐L. Chau

**Affiliations:** ^1^ Theory of Condensed Matter Group, Cavendish Laboratory, Department of Physics University of Cambridge Cambridge UK; ^2^ Bioinformatique Structurale, Institut Pasteur, CNRS URA 3528, CB3I CNRS USR 3756 Paris France

**Keywords:** GABARAP, GABA_A_ receptor, ionic conductance, electrostatic potential, ligand‐gated ion channels

## Abstract

We have performed fully atomistic molecular dynamics simulations of the intracellular domain of a model of the GABA_A_ receptor with and without the GABA receptor associated protein (GABARAP) bound. We have also calculated the electrostatic potential due to the receptor, in the absence and presence of GABARAP. We find that GABARAP binding changes the electrostatic properties around the GABA_A_ receptor and could lead to increased conductivity of chloride ions through the receptor. We also find that ion motions that would result in conducting currents are observed nearly twice as often when GABARAP binds. These results are consistent with data from electrophysiological experiments.

## 1 INTRODUCTION

The family of GABA_A_ receptors is responsible for the majority of fast neuronal inhibition in the mammalian central nervous system, and is a target of general anesthetics, benzodiazepines, barbiturates and neurosteroids. These pentameric proteins belong to the cys‐loop family of ligand‐gated ion channels that includes the nicotinic acetylcholine, glycine, and 5HT_3_ receptors. The GABA_A_ receptors are composed of five subunits arranged pseudosymmetrically around the central ion channel.^1^ The subunits, of which 19 have thus far been identified, are separated into classes based on their sequence similarity: there are six *α*‐subunits, three *β*, three *γ*, three *ρ*, and single representatives of *δ*, *ϵ*, *θ*, and *π*.^2^ The precise subunit isoform composition of the pentamer defines the recognition and biophysical characteristics of the particular receptor subtype. The most ubiquitous subtype, which accounts for approximately 30% of GABA_A_ receptors in the mammalian brain,^3^ contains two *α*_1_‐, two *β*_2_‐, and a single *γ*_2_‐subunit.^4^ The GABA_A_ receptors can be divided into three structural domains, the extracellular (EC) domain, the transmembrane (TM) domain, and the intracellular (IC) domain. When GABA binds to the GABA_A_ receptor, the central ion channel opens to let chloride ions through.^5^ This opening is quickly followed by a period of desensitization of the receptor.^6^

GABA_A_ receptors with a *γ*‐subunit are often associated with the GABA_A_‐receptor associated protein, GABARAP. This protein, first described by Wang et al.,^7^ consists of 117 amino acids and has a relative molecular mass of 13 900. Experimental work^7^, ^8^ shows that it binds to the intracellular domain of the *γ*_2_‐subunit of the GABA_A_ receptor. Its function is most probably twofold: anchoring the GABA_A_ receptor to the cytoskeleton, and modulating the function of the receptor. Amino acids near the N‐terminal of GABARAP could bind to tubulin,^9^ whilst the amino acids nearer the C‐terminal bind to the GABA_A_ receptor.^8^ Moreover, Chen et al.^10^ showed that GABARAP caused GABA_A_ receptor clustering, and clustered receptors exhibited lower affinity for GABA (EC_50_ increased from 5.74 ± 1.4 μM to 20.27 ± 3.8 μM), and they desensitized less quickly (the desensitisation time constant *τ* increased from 1 to 2 s). Luu et al.^11^ show that GABARAP binding increases the conductance of the GABA_A_ receptor from below 40 pS to above 50 pS, and the mean opening times from about 2 ms to about 6 ms.

Nevertheless, we still do not know how GABARAP binding changes the conductance of the GABA_A_ receptor. Previous study by Irwin et al.^12^ used experimental structures of the GABARAP and a modeled structure of the intracellular domain of the GABA_A_ receptor and performed docking, molecular dynamics simulations and inhomogeneous fluid solvation theory calculations to predict the interaction between GABARAP and the GABA_A_ receptor. We build on this study and use molecular dynamics simulations and electrostatic calculations to elucidate how GABARAP binding could increase the conductance of the GABA_A_ receptor.

## 2 METHODS

### 2.1 Molecular coordinates

In this research, we used the coordinates of a GABA_A_ receptor model from the study of Mokrab et al..^13^ This model used, as template, the nicotinic acetylcholine receptor (nAChR) structure from the study of Unwin,^14^ where five intracellular helices were resolved (Protein Data Bank code: 2BG9). This is the only structure of the GABA_A_ receptor, experimental or modeled, that includes part of the intracellular domain. The subunit composition of this receptor is (*α*_1_)_2_(*β*_2_)_2_ *γ*_2_. The intracellular helices are defined to be the following amino acids: *α*_1_‐subunit Lys 391–Asp 420, *β*_2_‐subunit His 421–Asp 450, *γ*_2_‐subunit Asp 413–Asp 442.

**FIGURE 1:**
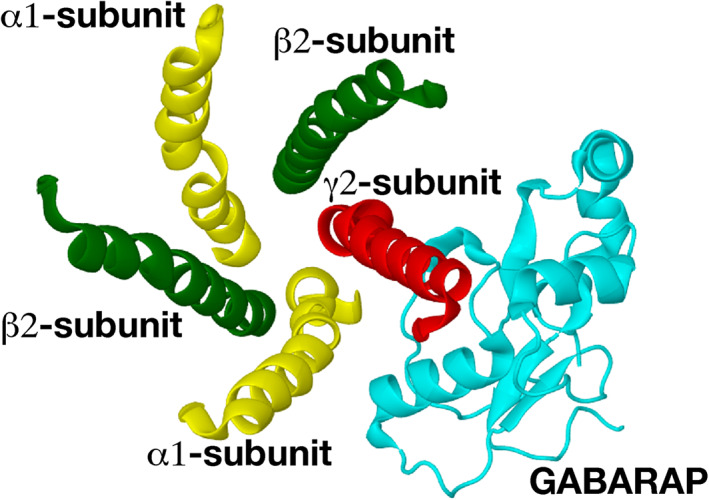
Model of the GABA_A_ receptor and a proposed docking pose of the GABARAP (1KOT model 15 dock 54a). The viewing direction is from the extracellular space towards the intracellular space. Only the intracellular helices of the GABA_A_ receptor are shown in this diagram. GABARAP is shown in cyan, the intracellular helix of the *γ*_2_‐subunit in red, that of the *α*_1_‐subunit in yellow and the helix of the *β*_2_‐subunit is shown in green

**FIGURE 2:**
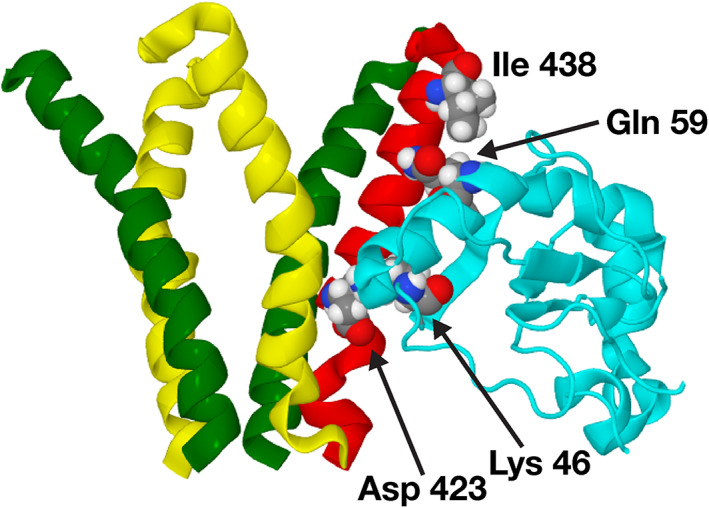
Side‐view of a model of the GABA_A_ receptor and a proposed docking pose of the GABARAP (1KOT model 15 dock 54a). Only the intracellular helices of the GABA_A_ receptor is shown in this diagram. GABARAP is shown in cyan, the intracellular helix of the *γ*_2_‐subunit in red, that of the *α*_1_‐subunit in yellow and the helix of the *β*_2_‐subunit is shown in green

For the GABARAP structure, we use dock 54a of structure 15 of the NMR solution structure (PDB code: 1KOT^15^) from previous study.^12^ Figure 1 shows the interaction between GABARAP and the GABA_A_ receptor intracellular pentahelix viewed from the extracellular space towards the cytoplasm. Figure 2 shows the interaction between GABARAP and the GABA_A_ receptor intracellular pentahelix from the side, with two amino acids from GABARAP and two amino acids from the pentahelix labeled.

### 2.2 Molecular dynamics simulation of GABARAP and intracellular helices

The systems were prepared using the CHARMM‐GUI freely available on the web.^16^ The molecular dynamics package NAMD 2^17^ was used in this study.

We took the pentahelix and immersed it in a solution consisting of 38658 water molecules, 110 K^+^ ions and 123 Cl^−^ ions in a periodic rhombohedral box measuring 108.9 Å by 109.0 Å by 108.8 Å which gives an ionic solution of concentration of about 0.15 M. We also took the pentahelix/GABARAP complex and immersed it in a solution consisting of 38024 water molecules, 108 K^+^ ions and 123 Cl^−^ ions in a periodic rhombohedral box of the same dimensions which gives an ionic solution of concentration of about 0.15 M. These protein molecules are charged, so unequal numbers of cations and anions are included to render the final systems electrically neutral. In both cases, the protein is at least 10 Å from any part of its image in the next periodic box.

**FIGURE 3:**
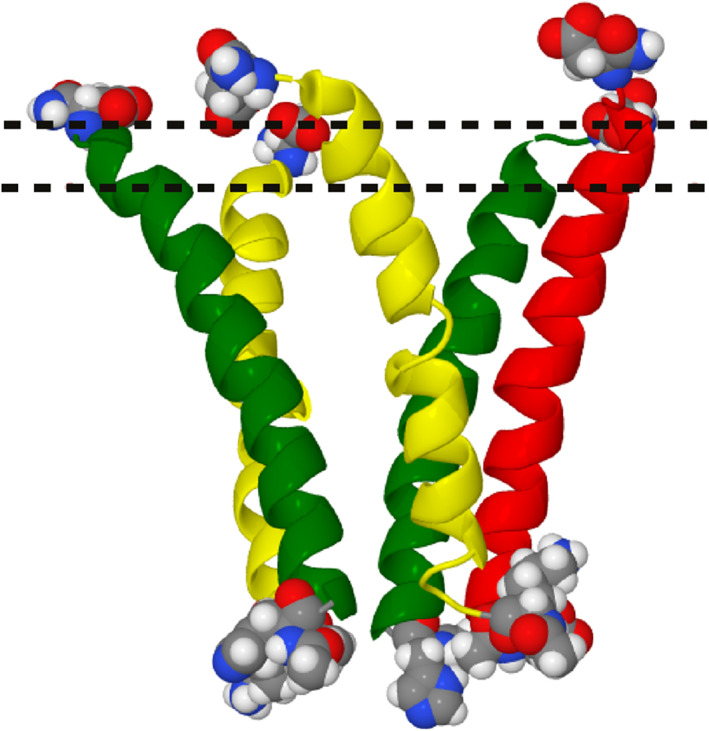
Side‐view of a model of the GABA_A_ receptor intracellular domain; the amino acids which form the vertices of the convex hull are shown in CPK models. The intracellular helix of the *γ*_2_‐subunit is shown in red, that of the *α*_1_‐subunit in yellow and that of the *β*_2_‐subunit is shown in green. The top dashed line is where *z* = 20 Å and the bottom dashed line is where *z* = 15 Å

We used the CHARMM potential for all our simulations.^18^ Each system was minimized for 10 000 steps with all the protein atoms frozen. Molecular dynamics at 310 K was initialized for 10 000 time‐steps of 0.1 fs each, with all main‐chain nitrogen atoms frozen. Langevin dynamics was applied; the thermostat was set with a time constant of 1 ps^−1^, and the barostat set with a piston decay time of 10 ps and a piston period of 20 ps. The van der Waals cut‐off was 12 Å, and Ewald summation was used for the electrostatic interactions arising from the cell's periodic images. The time‐step was lengthened to 2 fs over 30 000 time‐steps, during which period all main‐chain nitrogen atoms of the three helices were tethered with a force constant of 2 kJ/mol/Å^2^. These helices are part of a large protein and the helical structures are stabilized by neighboring structures, some of unknown configuration. In this study, we included only the helices and so to stabilize them, we imposed the tethers. A 50‐ns equilibration was carried out on the initialized system, followed by a data collection period of 100 ns. Equilibration was confirmed by a stable r.m.s. deviation from the starting structure and, in the case of the pentahelix/GABARAP complex, a stable intermolecular distance. Configurations were output every 20 ps. A convex hull was created using the following 10 amino acids as vertices (they are at the end points of the five intracellular helices) using a previously developed method^19^: chain A (*β*_2_‐subunit) His 421 and Asp 450, chain B (*γ*_2_‐subunit) Asp 413 and Asp 442, chain C (*α*_1_‐subunit) Lys 391 and Asp 420, chain D (*β*_2_‐subunit) His 421 and Asp 450 and chain E (*α*_1_‐subunit) Lys 391 and Asp 420. These 10 amino acids are shown in Figure 3; the vertices on the membrane side lie (upper side of the diagram) in the plane where *z* ∼ 20 Å and those on the intracellular side (lower side of the diagram) lie in the plane where *z* ∼−20 Å. The number of Cl^−^ ions inside this convex hull was determined using a previously developed method^20^ and counted for every configuration.

We tracked the trajectories of the ions to locate movements which are similar to Cl^−^ ion movements when the GABA_A_ receptor is conducting naturally. We define a “natural” ion movement where the Cl^−^ ion moves into the convex hull from the membrane side across the plane where *z* ∼ 20 Å and where it exits from one of the five side portals at positions where *z* < 15 Å (see Figure 3); previous experiments show that these side portals are the exit routes for ions.^21^

### 2.3 Evaluation of electrostatic potential

We calculated the electrostatic potential around the GABA_A_ receptor intracellular pentahelix, in the absence and presence of GABARAP. From the 100‐ns data production run of the molecular dynamics simulation, we took a configuration at every 10 ns to obtain 10 configurations. The water molecules and ions were removed from these configuration and, for each configuration, we calculated the electrostatic potential due to the CHARMM partial charges^18^ on the protein atoms using simple Coulombic interactions; the dielectric constant was taken as one and nonperiodic boundary conditions were applied. We then averaged the potential over the 10 configurations and compared them in the absence and presence of GABARAP.

## 3 RESULTS

### 3.1 Molecular dynamics simulations

**FIGURE 4:**
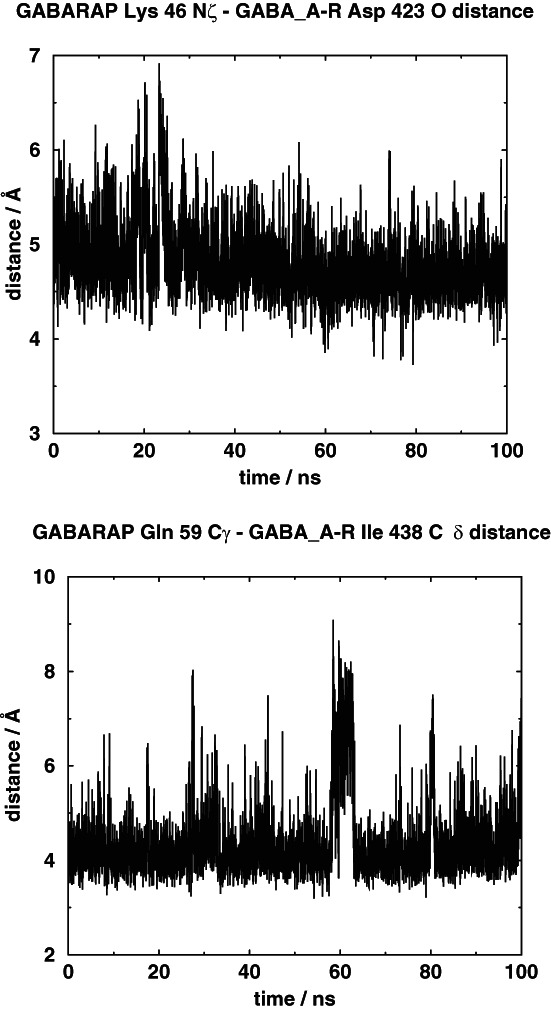
The upper panel shows the distance between the GABARAP Lys 46 N*ζ* atom and the GABA_A_ receptor *γ*‐subunit Asp 423 main‐chain oxygen atom and the lower panel shows the distance between the GABARAP Gln 59 C*γ* atom and the GABA_A_ receptor *γ*‐subunit Ile 438 C*δ* atom

The GABA_A_ receptor intracellular pentahelix atoms moved little during the course of the 100 ns data collection simulation, as they were tethered. GABARAP was not tethered, but it stayed in close proximity of the pentahelix. The volume enclosed by the amino acids at the end points of the intracellular helices were calculated using a previously developed method^22^ and it remained stable at a value of about 27 nm^3^ during the course of the simulation (data not shown). We measured the distances between, respectively, GABARAP Lys 46 N*ζ* and the GABA_A_ receptor *γ*‐subunit Asp 423 main‐chain O, and GABARAP Gln 59 C*γ* and the GABA_A_ receptor *γ*‐subunit Ile 438 C*δ* and used these distances as indicators of the distance between these two proteins. The results are shown in Figure 4. It can be seen that the distances are relatively constant, which shows that the complex was stable throughout the data collection period.

**FIGURE 5:**
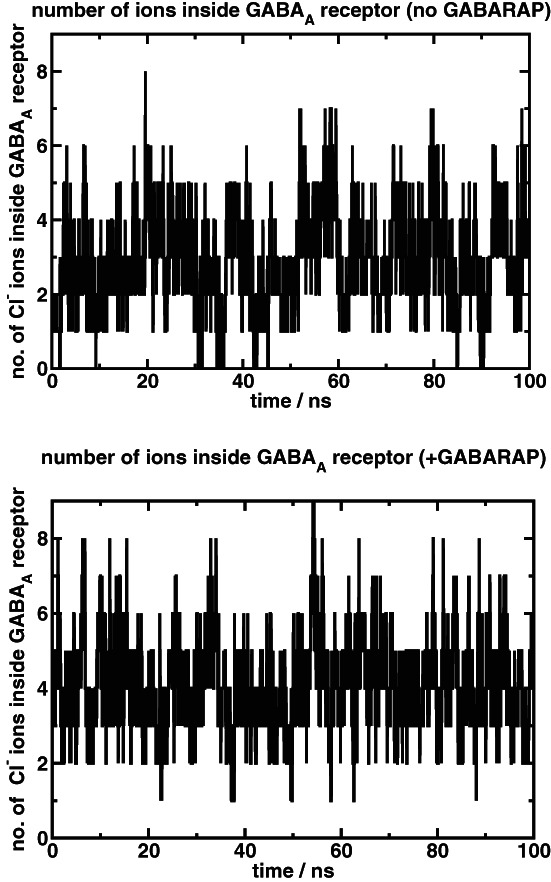
Number of ions inside the convex hull in the absence of GABARAP (upper panel) and in the presence of GABARAP (lower pane)

Figure 5 shows the number of ions inside the convex hull enclosed by the pentahelix. There is an average of 2.8 Cl^−^ ions inside the convex hull in the absence of GABARAP, but on GABARAP binding this increases to 4.0 Cl^−^ ions. The number of ions in the pentahelix changes over time as the ions move in and out of the pentahelix, which is a pre‐requisite for conduction. Moreover, in the presence of GABARAP, the average number of ions in the pentahelix is about 40% higher than in the absence of GABARAP, suggesting that more ions may be moving through the channel. In the absence of GABARAP, there are configurations when the channel has no ions, at which point it cannot be conducting ions.

**FIGURE 6:**
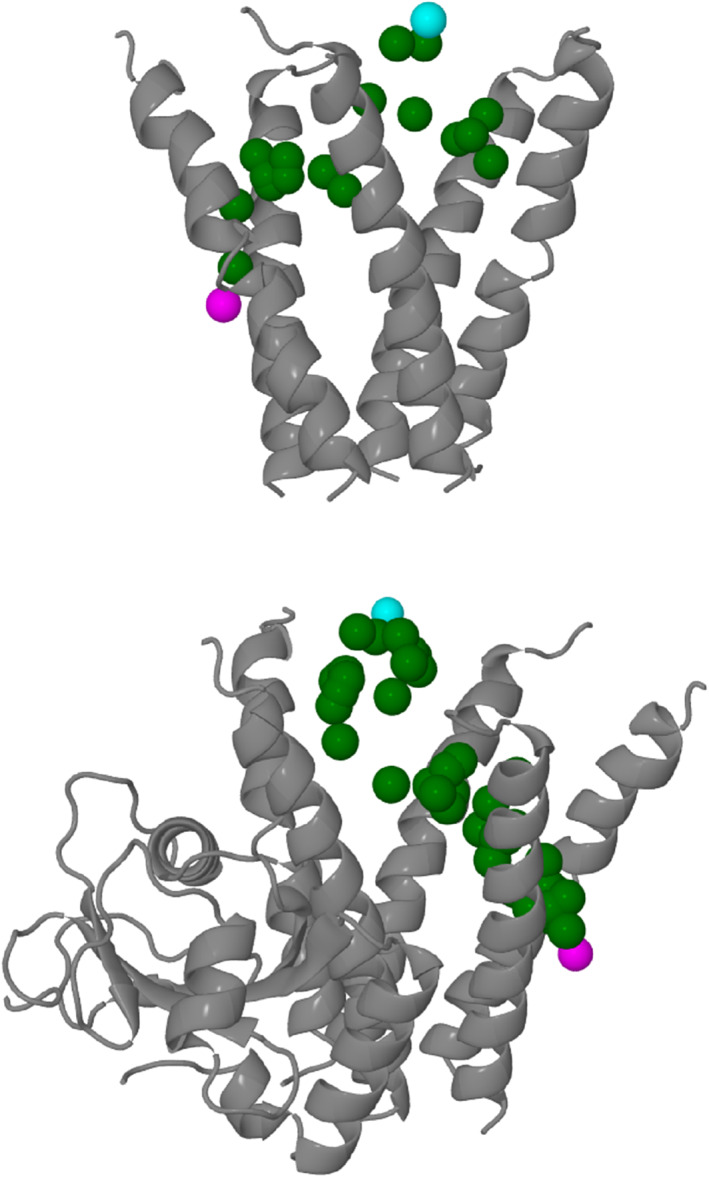
The top diagram shows a Cl^−^ ion entering the convex hull from the membrane side and exiting it from one of the five portals on the side at a level where *z* = −4.4 Å. The protein is shown in grey, the Cl^−^ ions in green except for the starting position (in cyan) and the finishing position (in magenta). Each position of the Cl^−^ ion is 20 ps apart. The bottom diagram shows a similar event but in the presence of GABARAP. Both trajectories are under 1 ns

We observed ions moving from the membrane side of the convex hull, through the hull of the pentahelix, then exiting from one of the five portals on the side, at positions where *z* < 15 Å. Figure 6 shows examples of such movements. Note that these ion passage trajectories usually last <1 ns, and they are short events on the timescale of the simulation. We observed 32 such events when GABARAP was absent but 60 such events when GABARAP was present during the 100 ns molecular dynamics simulations.

### 3.2 Electrostatic potential

**FIGURE 7:**
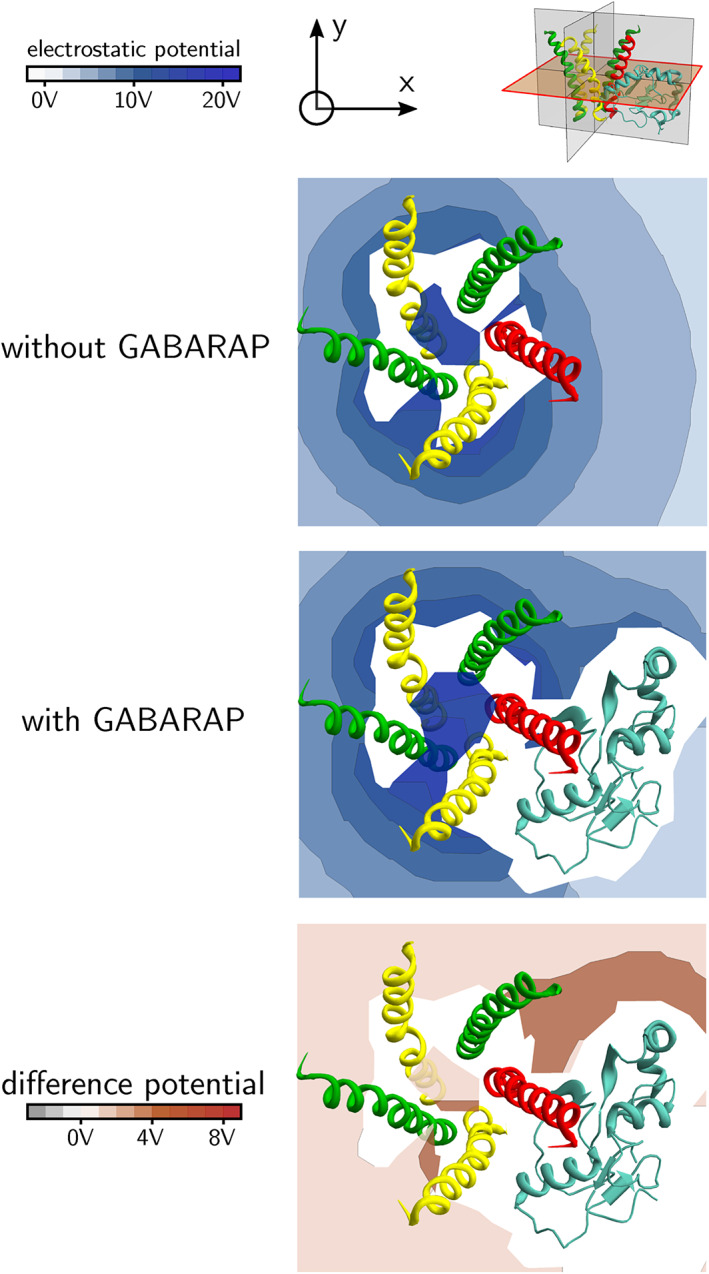
Electrostatic potential around GABA_A_ receptor intracellular helices. The top panel shows, respectively from left to right, the electrostatic potential scale, the axes of the system and a diagram showing the transverse plane. The second panel shows the electrostatic potential on the plane in the absence of GABARAP, and the third panel shows the potential in the presence of GABARAP. The bottom panel shows the difference in electrostatic potential between the two cases. The *α*_1_‐subunit is shown in yellow, the *β*_2_‐subunit is shown in green and the *γ*_2_‐subunit is shown in red

We visualize the electrostatic potentials due to the protein(s) by displaying the values on different planes using a color‐coded scheme. In Figure 7, the electrostatic potential is displayed on a plane perpendicular to the central axis of the receptor. In the absence of GABARAP, the electrostatic potential is more positive in the region around the *β*_2_‐subunits. In the presence of GABARAP, there is a finger‐like “extension” of more positive electrostatic potential through the slit made by the *β*_2_‐ and *γ*_2_‐subunits next to the bound GABARAP. The region over which the electrostatic potential increases is largely outside the pentahelix.

**FIGURE 8:**
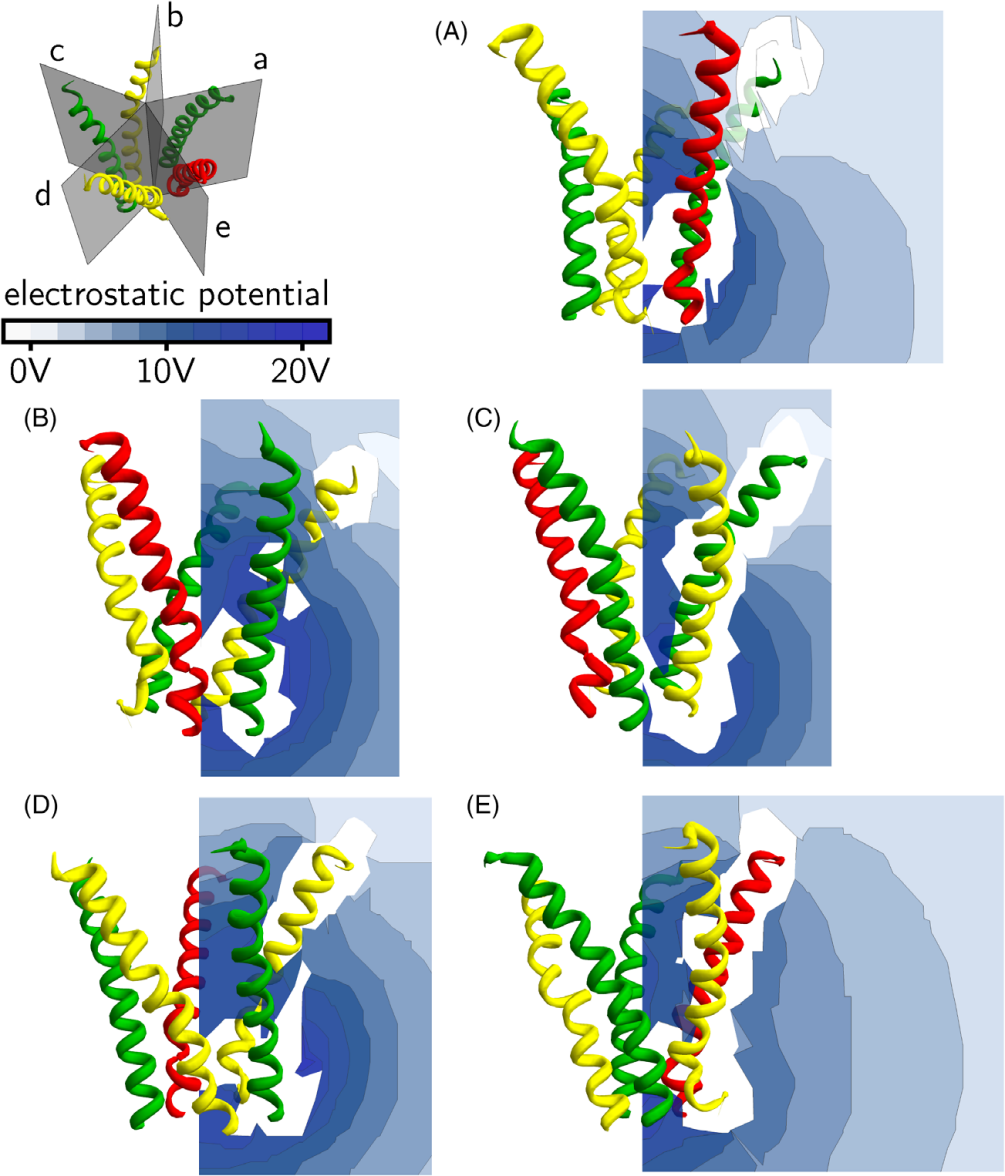
Electrostatic potential around GABA_A_ receptor intracellular helices. The *α*_1_‐subunit is shown in yellow, the *β*_2_‐subunit is shown in green and the *γ*_2_‐subunit is shown in red. The top left panel shows five planes, each cutting through one of the five slits formed by the helices. The electrostatic potential due to the protein alone is calculated and displayed in a color‐coded scheme. Panels (A–E) show the electrostatic potential on the five planes

**FIGURE 9:**
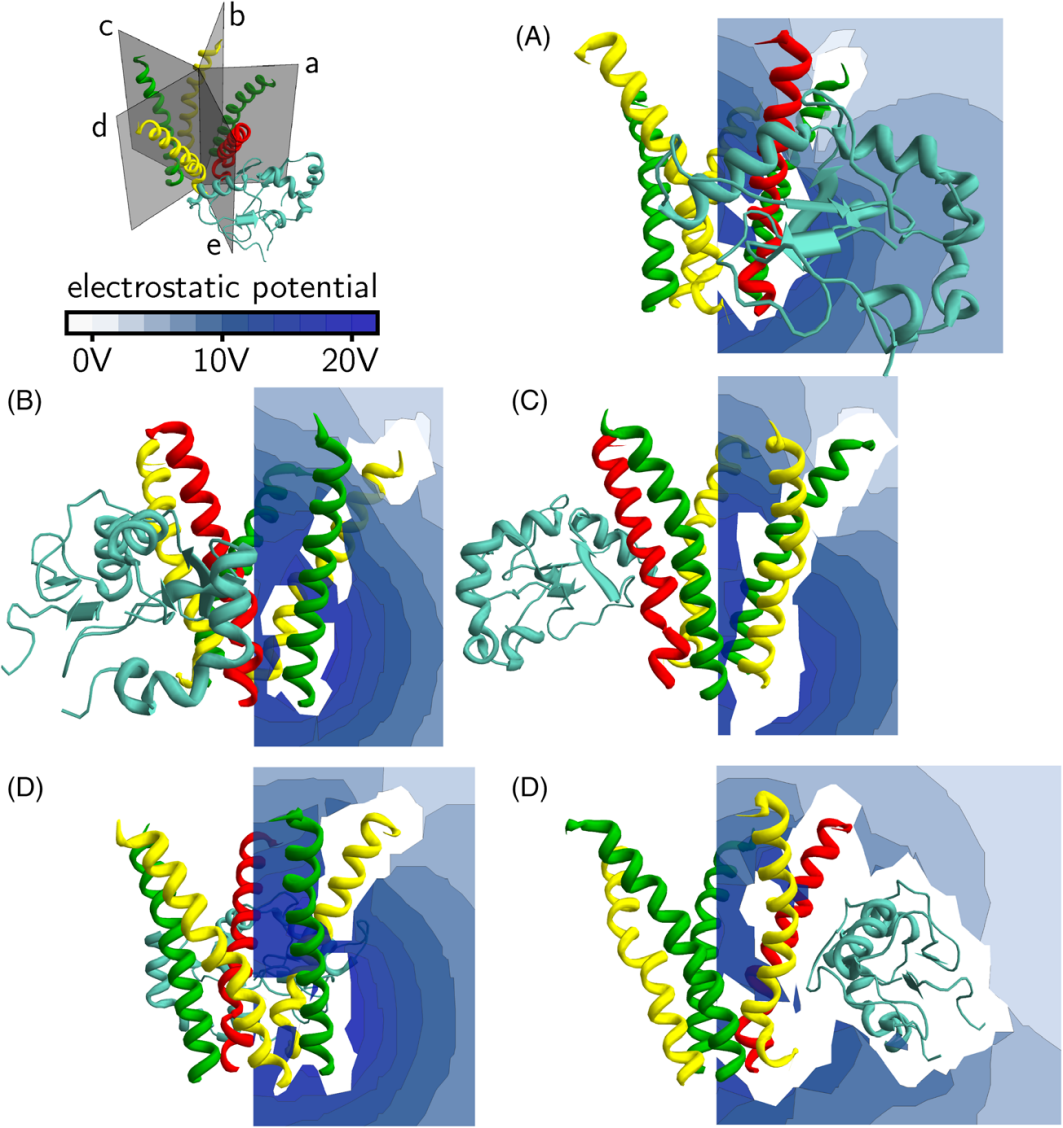
Electrostatic potential around GABA_A_ receptor intracellular helices and GABARAP. The *α*_1_‐subunit is shown in yellow, the *β*_2_‐subunit is shown in green, the *γ*_2_‐subunit is shown in red and GABARAP shown in cyan. The top left panel shows five planes, each cutting through one of the five slits formed by the helices. The electrostatic potential due to the proteins is calculated and displayed in a color‐coded scheme. Panels (A–E) show the electrostatic potential on the same five planes as in Figure 8

**FIGURE 10:**
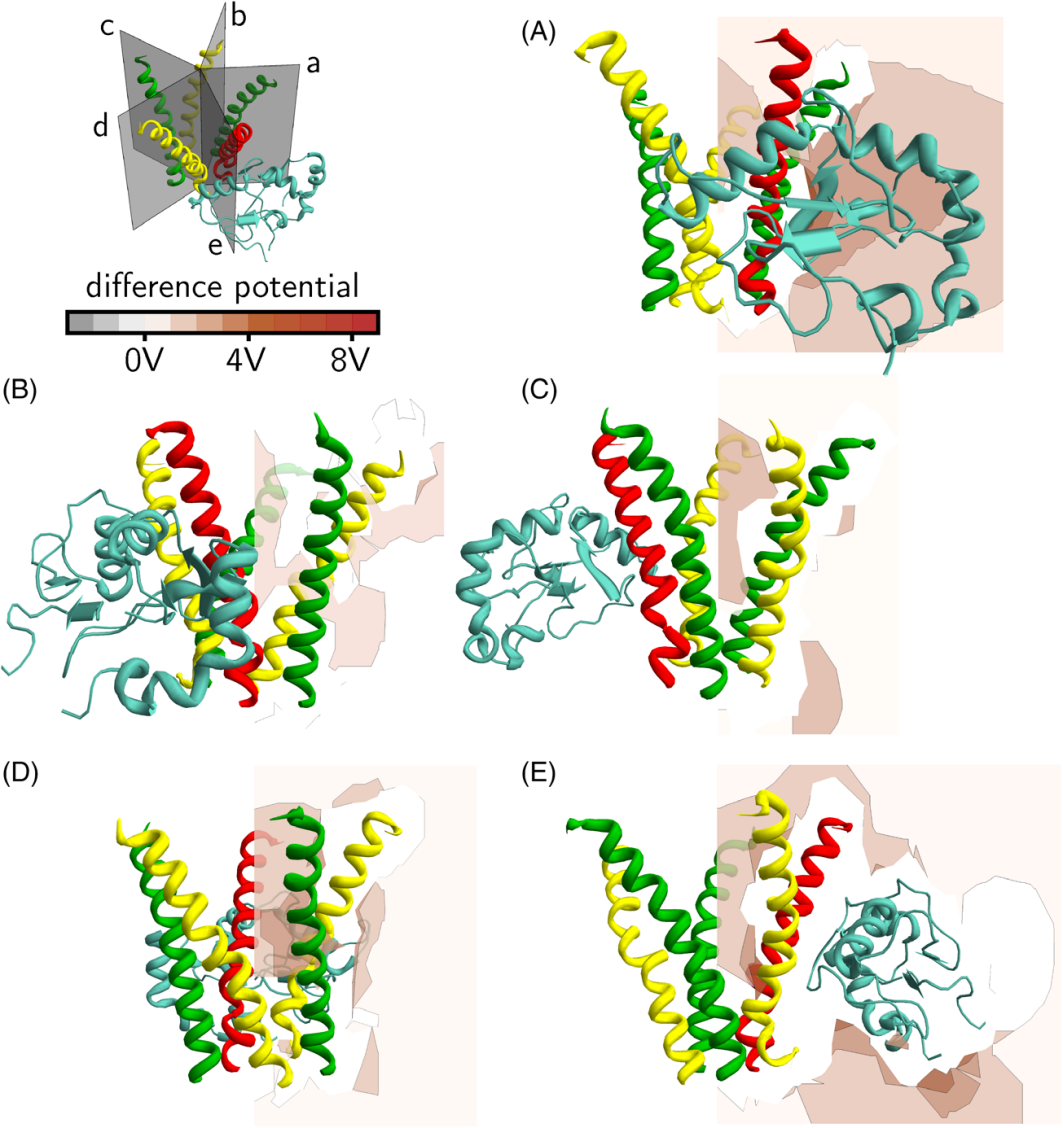
Difference electrostatic potential (the difference in electrostatic potential between the case where GABARAP is absent and the case where GABARAP is present) around GABA_A_ receptor intracellular helices. The *α*_1_‐subunit is shown in yellow, the *β*_2_‐subunit is shown in green, the *γ*_2_‐subunit is shown in red and GABARAP shown in cyan. The top left panel shows five planes, each cutting through one of the five slits formed by the helices. The difference electrostatic potential due to the protein alone is calculated and displayed in a color‐coded scheme; a positive difference means that the electrostatic potential in the presence of GABARAP is more positive than in its absence. Panels (A–E) show the difference electrostatic potential on the same five planes as in Figure 8

In Figures 8 and 9, the electrostatic potential is displayed on five planes through each of the five slits formed by the GABA_A_ receptor intracellular helices. In Figure 8, the electrostatic potential due to the intracellular helices alone is displayed. In Figure 9, the electrostatic potential due to the intracellular helices and GABARAP is shown. It can be seen that the effect of GABARAP on the electrostatic potential in planes (B) and (C) is small. However, in planes (A), (D) and (E), the electrostatic potential is more positive in the region outside the intracellular helices (Figure 9). To make it easier to visualize these changes in electrostatic potential, we plot the difference potential in Figure 10; this is the difference in electrostatic potential between the case where GABARAP is absent and the case where GABARAP is present. A positive difference means that the electrostatic potential in the presence of GABARAP is more positive than in its absence. It can be seen from Figure 10 that most regions outside the pentahelix become electrostatically more positive due to the presence of GABARAP, but some regions towards the cytoplasmic end inside the pentahelix become more negative. We suggest that this increase in electrostatic potential outside the receptor with a concomitant decrease in potential inside the receptor leads to the increase in Cl^−^ ion conductance.

## 4 DISCUSSION

Cys‐loop ligand‐gated ion channels often interact with cytoplasmic proteins, and this interaction serves many purposes, amongst them the clustering of ion channels and the modulation of channel function.

For example, the muscle nicotinic acetylcholine receptor (nAChR) interacts with the cytoplasmic protein rapsyn. Rapsyn has a molecular weight of about 43 000,^23^ and electron microscopy showed that the nAChR are interconnected by rapsyn dimers. Up to three rapsyn dimers can contact each nAChR in specific regions in the nAChR intracellular domain.^24^ This tight network probably anchors the nAChR in the plane of the cell membrane and allows nAChR to be concentrated at the neuromuscular junction motor end‐plate.^24^

Another example is gephyrin. This protein was first identified as a bridge between the glycine receptor and tubulin.^25^ Sola et al.^26^ co‐crystallized a segment of the glycine receptor *β*‐subunit and a partial dimer of the cytoplasmic protein gephyrin (Protein Data Bank code: 1T3E). They resolved the structure of a pentapeptide portion of the glycine receptor *β*‐subunit and the gephyrin domain E dimer. These scientists proposed a network of gephyrin molecules linking the glycine receptors.

Gephyrin also interacts with the GABA_A_ receptor through the receptor *α*_2_‐subunit^27^ and *α*_3_‐subunit.^28^ It is unclear if gephyrin binds the *α*_1_‐subunit of the GABA_A_ receptor; some experiments failed to show any interaction,^29^ but others showed a weak interaction.^30^ Maric et al.^31^ co‐crystallized segments of the *α*_3_‐subunit of the GABA_A_ receptor with segments of gephyrin, and identified the undecapeptide T^367^FNIVGTTYPIN^381^ from the GABA_A_ receptor as important for interaction with gephyrin. They showed that there were similarities between the binding of the GABA_A_ receptor and of the glycine receptor to gephyrin: in particular, T^367^FNIVGTT^374^ from the GABA_A_ receptor, and F^398^SIVGSL^404^ from the glycine receptor *β*‐subunit adopted similar conformations.

In addition to gephyrin, the GABA_A_ receptor also interacts with collybistin; there are two types of collybistin, which consist of 413 and 493 amino acids, respectively.^32^ Saiepour et al.^29^ showed that collybistin interacted with the intracellular domain of the *α*_2_‐subunit of the GABA_A_ receptor, and its binding site for the *α*_2_‐subunit overlapped that for gephyrin. Collybistin was later shown to be important for clustering gephyrin and the GABA_A_ receptor.^33^

The GABA_A_ receptor also interacts with GABARAP. GABARAP binds specifically to the *γ*_2_‐subunit of the GABA_A_ receptor. Binding of GABARAP to the GABA_A_ receptor causes receptor clustering,^10^, ^34^ so some of its functions are similar to gephyrin and collybistin. However, GABARAP is unique in that its binding also causes the conductance of the GABA_A_ receptor to increase from about 30 pS to 40 pS–60 pS, and the mean channel opening times from about 2 ms to about 6 ms.^11^ It thus appears that gephyrin has more general actions on both the GABA_A_ receptor and the glycine receptor, and that the action of gephyrin and collybistin appear to be confined to receptor clustering. The action of GABARAP is more specific to the GABA_A_ receptor, and, in addition to receptor positioning, it also modulates the electrophysiology of this ion channel.

The GABA_A_ receptors in neurons have different ion channel properties from recombinant receptors.^35^ Luu et al.^11^ show that GABA_A_ receptor conductances in neurons is similar to that obtained from recombinant receptors associated with GABARAP. GABARAP is thus of importance in physiological functioning of the GABA_A_ receptor in the central nervous system, and this underlies the importance of understanding the physiological role of the intracellular domain of this receptor.

In this study, we used a simplified system of a modeled GABA_A_ receptor consisting only of its intracellular domain and studied its electrostatic properties in the absence and presence of GABARAP. Our results show that GABARAP increases the electrostatic potential of the region around and outside the intracellular domain; this is consistent with increased Cl^−^ conductance. Our results also show that the number of ions in the pentahelix varies significantly in time as they move in and out of the pentahelix, which is a pre‐requisite for conduction. Moreover, in the presence of GABARAP, the average number of ions in the pentahelix is more than 40% higher, suggesting that more ions may be moving through the channel. In the absence of GABARAP, there are configurations when the channel has no ions, at which point it cannot be conducting ions. Further analysis shows that ion movements through the convex hull of the pentahelix similar to “natural” conducting currents are almost twice as frequently observed in the presence of GABARAP. In both cases the number of these conduction events observed during a 100‐ns period was over 10 times higher than the average number of ions in the convex hull, and there was no evidence of a long‐term increase or reduction in the number of ions inside the hull.

Previous experimental results^11^, ^35^ and our findings in this article show that GABARAP binding to the GABA_A_ receptor increases the receptor channel conductance. However, the exact role of GABARAP is thrown into doubt by recent experiments. Everitt et al.^34^ suggest that GABARAP is not involved in altering GABA_A_ receptor conductance. Tierney^36^ suggests that adjacent GABA_A_ receptors interact via their solitary *γ*_2_‐subunit MA helices; the ionic conductance is thus increased by this interaction. However, in the suggested mechanism, the *γ*_2_‐subunit of one GABA_A_ receptor swings out to interact with the *γ*_2_‐subunit of another receptor, which involves a large structural change. These results seem to contradict previous experimental findings.^11^, ^35^

To define the interaction between GABARAP and the GABA_A_ receptor in greater detail and to understand how GABARAP changes the receptor structure and function would require high‐resolution structures of the GABA_A_ receptor with an intact intracellular domain, in the absence and presence of GABARAP. This should then be accompanied by electrophysiology experiments where, ideally, the behavior of three membrane patches are compared: the first patch contains one single active GABA_A_ receptor and no GABARAP, the second patch contains one single active GABA_A_ receptor with GABARAP, and the third patch contains two or more active GABA_A_ receptors. In the third patch, the interaction between the individual GABA_A_ receptors can be disrupted using different peptides to define the interaction between the different molecules. This kind of system would allow us to examine in detail the apparent contradiction in previous experimental results^11^, ^34^, ^35^, ^36^ and arrive at a better understanding of the function of GABARAP.

## CONFLICT OF INTEREST

The authors declare no conflict of interest.

### PEER REVIEW

The peer review history for this article is available at https://publons.com/publon/10.1002/prot.26241.

## Data Availability

"The simulation data are available on request from the corresponding author (pc104@pasteur.fr) but in due course they will be put on a server where they can be downloaded by readers."
